# Electrolyzed Water Generated On-Site as a Promising Disinfectant in the Dental Office During the COVID-19 Pandemic

**DOI:** 10.3389/fpubh.2021.629142

**Published:** 2021-04-30

**Authors:** Ra'fat Ibrahim Farah, Sanaa Najeh Al-Haj Ali

**Affiliations:** ^1^Department of Prosthetic Dental Sciences, College of Dentistry, Qassim University, Buraydah, Saudi Arabia; ^2^Department of Orthodontic and Pediatric Dentistry, College of Dentistry, Qassim University, Buraydah, Saudi Arabia

**Keywords:** COVID-19, disinfectant, electrolyzed water, microbiology, SARS-CoV-2

## Abstract

Electrolyzed water is a safe, broad-spectrum bactericidal and viricidal agent, which can be used as a potent and effective alternative disinfectant in case of supply shortages. This report describes the on-site production of slightly acidic electrolyzed water (EW) from diluted salt solution and vinegar at a dental office using a portable EW generator unit. Such measures can ensure the safe continuity of important dental service provision for our patients during the coronavirus disease 2019 (CoVID-19) pandemic.

## Introduction

More than a year has passed since the World Health Organization (WHO) characterized coronavirus disease 2019 (CoVID-19) as a pandemic (1), which has increased worldwide demand for disinfectants and sanitizers in the medical and health care sectors, as well as for other institutional and individual use. Manufacturers have been unable to fully cope with this increased demand. Disinfectants are very important in dental offices to ensure safe environments for dentists, staff, and patients. Identifying alternative sources of safe and effective disinfectants for use in emergencies, such as the current supply crisis, will enable the safe continuity of dental service provision for our patients.

Electrolyzed water (EW) is a chlorine-based disinfectant that can be relatively easily made on-site via the electrolysis of pure table salt (sodium chloride, NaCl) solution, utilizing one of many commercially available electrolysis devices (2). Hypochlorous acid (HOCl) is the primary antibacterial agent in such in-office-generated EW disinfectants, which can therefore be used under the regulations for HOCl disinfectants already approved by the US Environmental Protection Agency (EPA) as appropriate for use against SARS-CoV-2 (3–5).

The first discovery and development of EW can be tracked to more than a century ago (6). In the 1950s, EW was applied in general agriculture. In the late 1970s and early 1980s, the use of EW was proposed in drinking water disinfection and wastewater treatments. Next, during the 1980s, EW was commercially introduced in the food industry in Japan as sanitation water, stored in an automatic dispenser, for use in food processing in the soda industry. Since then, EW has attracted the attention of many researchers and numerous studies have reported the effectiveness of different types of EW as disinfectants in the food industry (7). At the beginning of this century, EW was officially approved by regulatory authorities in Japan and the U.S. for use as a sanitizer in the food industry (3). With recent developments in technology, industries have improved technologies to increase the effectiveness of EW; since 2010, many innovative companies have appeared on the market and EW generators have become available to individuals and small businesses (7, 8). Furthermore, EW was also proposed for use in medicine, to reduce bacterial counts in wound care, and to disinfect the skin around the eyes in ophthalmology (9, 10). In dentistry, EW has been effective against oral bacteria. It significantly reduced five major periodontal pathogens when used as a mouthwash and for toothbrush disinfection, even when EW was produced via electrolysis of drinking water, without adding salt (11). EW also has antifungal activity and can reduce the levels of *Candida albicans* biofilm on denture resins; a denture storage and disinfection device based on EW was recently developed (12). Furthermore, running EW through dental unit waterlines, even for a short period, was found to be effective in minimizing microbial biofilm contamination in the waterlines; and its use reduced the count of viable bacteria to negligible levels in coolant water spray from handpieces and three-way syringes, thus ensuring output water of good microbiological quality (13, 14). EW has recently been proposed as the disinfectant of choice for coronaviruses in an oral and maxillofacial surgery office (15). Official criteria have been published describing the applications of EW in Japan, the US, the EU, and China in many fields (6).

EW can be generated in three forms: alkaline/basic EW, acidic EW, and neutralized EW. However, the most utilized form, the efficacy of which has been widely investigated, is acidic EW (6, 16). The potent antimicrobial properties of acidic EW were attributed by many previous studies to be a result of free available chlorine (FAC) oxygen radicals, and reactive hydrogen, as well as low pH or the interaction/combination of these factors (17). FAC is essentially dissolved chlorine gas (Cl_2_); hypochlorous acid and/or hypochlorite ions (ClO^−^) are often referred to as “free available chlorine.” The primary ingredient of EW in a slightly acidic solution at 20°C is HOCl, as HOCl represents 90% of the FAC at pH 7; however, HOCl content decreases with increasing pH such that at pH 7.6 HOCl represents 50% and at pH 8.6 only 10% of the FAC, the predominant species changing to ClO^−^ (18). HOCl is considered the most active form of chlorine in this context. It is the strongest oxidant, with an oxidation-reduction potential (ORP) of +800 to +1100 mV, and has 80-fold more antimicrobial activity than ClO^−^ (19). EW can act on a wide variety of biomolecules, including DNA, RNA, fatty acid groups, cholesterol, and proteins. Acidic EW's proposed modes of action against microorganisms include destroying microbial membranes, chlorination by forming chloramines, decarboxylation of amino acids, reactions with nucleic acids, and unbalanced metabolism after the destruction of key enzymes (20–22).

EW has been extensively used in the food industry and the agriculture and food science and technology literature contain many relevant studies (2, 17). However, few studies have investigated EW in the dental literature. This report aims to fill this gap by describing the generation of a slightly acidic EW disinfectant using a commercially available portable EW generation device and verifying its FAC and acidity (pH) as indictors of disinfectant potency/efficacy. The results presented here may open the door to more applications and further studies on this potent, safe, and on-site-producible solution as an alternative disinfectant in dentistry, especially during shortages in other disinfectant supplies.

## Report

The acidic EW production method used in this evaluation was as follows. Pure non-iodized table salt (pure NaCl) was mixed with distilled water at 2 g per liter to produce a 0.2% salt solution, to which 5 ml of distilled white vinegar (5% acidity, pH 2.5) was added. This will make the solution slightly acidic (pH 4–6), which will shift the equilibrium (in the equation: ClO^−^ + H^+^ ⇌ HOCl) toward the more potent antimicrobial HOCl (19). Pour the solution into the electrolysis water unit turn on the unit and wait until electrolysis has completed (usually 10 min) to generate the disinfectant solution.

As an alternative to making the 2% salt solution specified above, either 0.45 or 0.9% sodium chloride normal saline solutions could be used. However, lower NaCl concentrations will reduce the HOCl concentration generated, which is directly proportional to the antimicrobial effect and inversely proportional to corrosion and biologic compatibility (4, 16).

**Figure 1:**
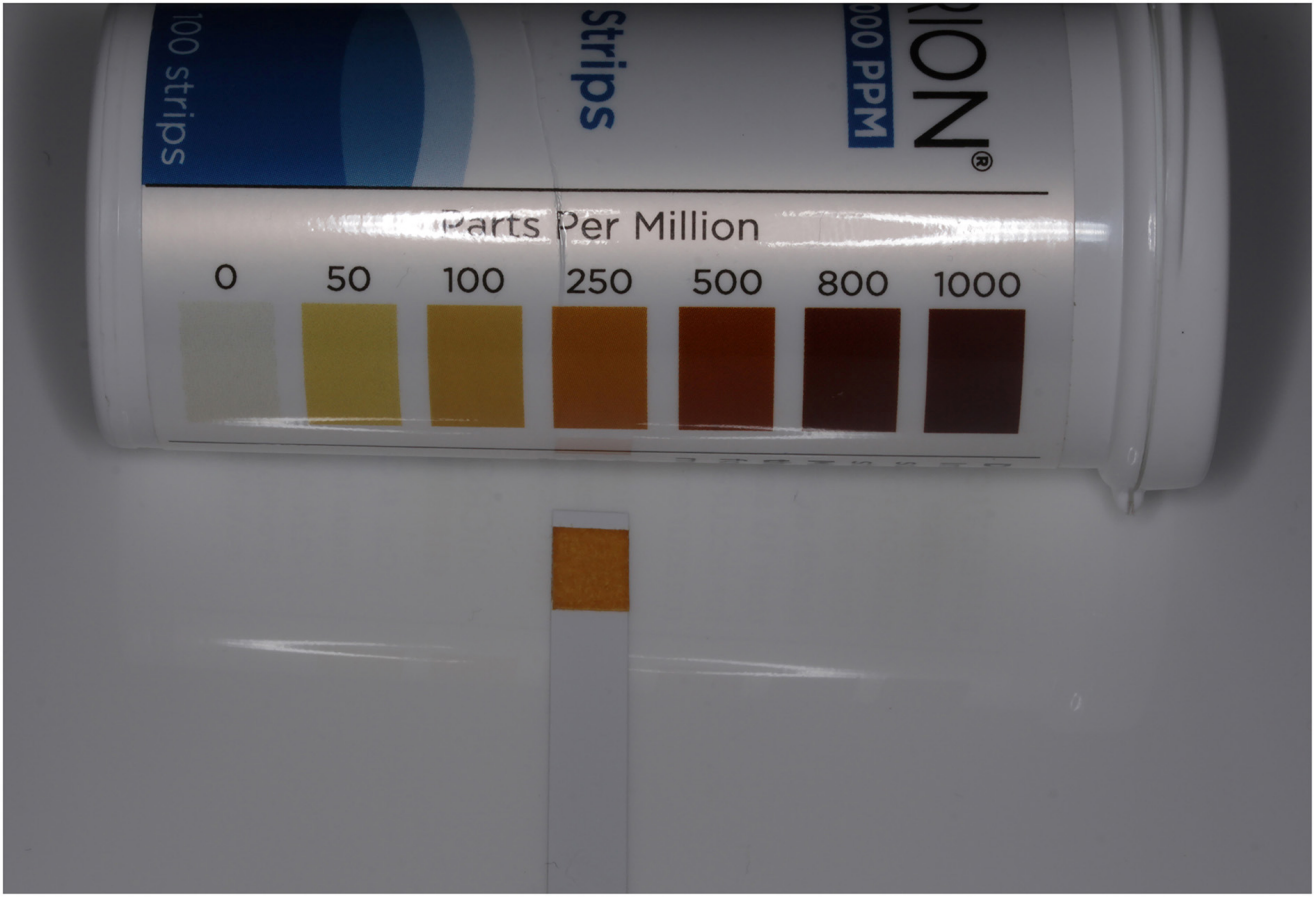
Chlorine test strip showing FAC concentration (250 ppm) resulting from 0.2% salt solution.

**Figure 2:**
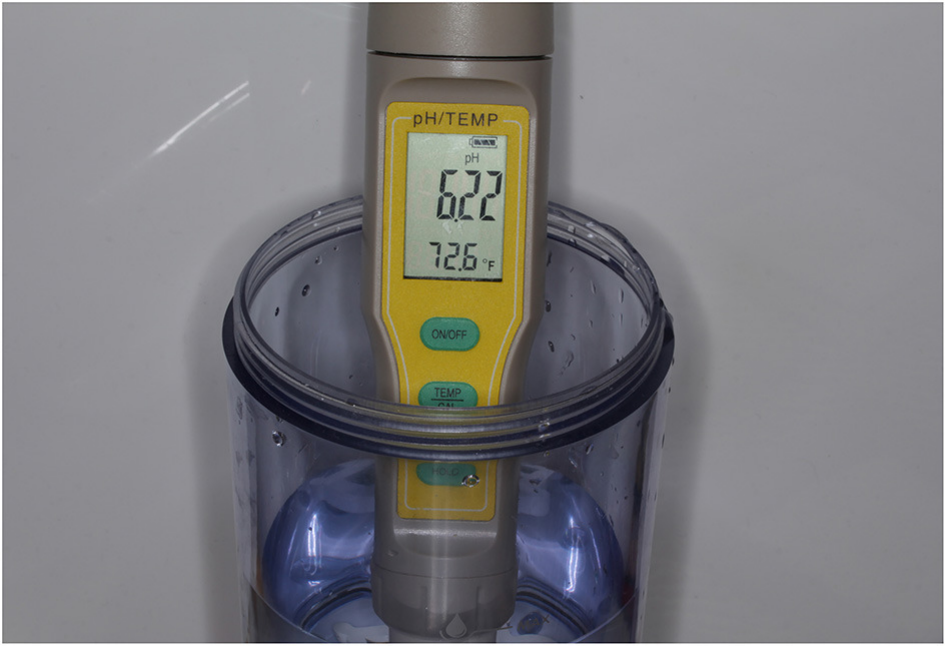
Measurement of solution acidity using a digital pH meter, which indicates a slightly acidic solution (~pH 6.5).

The concentration of FAC in the resulting solution was measured by dipping a chlorine test strip (Hydrion Chlorine indicator strips, Micro Essential Laboratory) for 1 second into it, blotting the strip with a paper towel, and then comparing the resulting color with the matching color chart code, which identified the chlorine concentrations in parts per million (ppm) (Figure 1). The pH of the solution was measured using a digital pH tester (pH/Temperature meter, Yieryi). The target pH was 5–6.5 (Figure 2). Alternatively, a pH test strip could be used.

For clinical use, the solution can be sprayed onto the surface to be disinfected or used as a hand sanitizer. EW at HOCl concentration of 200 ppm is effective in inactivating a variety of viruses, including coronaviruses, noroviruses, and other enteric viruses (15).

Recent studies (4, 15, 23) reported the effectiveness of EW with HOCl concentrations as low as 100 ppm against SARS-CoV-2, and many HOCl-based products have been developed that meet the EPA criteria for use against SARS-CoV-2 (5). Moreover, previous studies (24, 25) showed that slightly acidic EW and neutral EW generated in single-cell units (without diaphragm), similar to that mentioned in this report, were effective against difficult-to-kill viruses/pathogens (such as non-enveloped viruses (e.g., norovirus) (26). However, the rapid viricidal effect of any EW disinfectant depends on the FAC (specifically HOCl) concentration; at a lower FAC (such as in old solutions that have been in contact with the atmosphere for a long period), the disinfectant ability of the solution is lost and the EW can no longer effectively inactivate SARS-CoV-2 (4).

## Discussion

The science behind the on-site generation of EW is the well-understood process of electrolysis in a saline solution. Conventionally, the electrolysis unit is composed of a two-cell chamber (anode and cathode) separated by a membrane or a diaphragm. By passing a low level of direct current (10–20 V) into the diluted salt solution, the anode cell attracts negatively charged ions (Cl^−^), which react with dissociated water molecules (H^+^ and O_2_) to form hypochlorous acid (HOCl, the anolyte). This creates an acidic EW, with a pH of 2.5–3.5. The cathode attracts positively charged ions (Na^+^), which react with hydroxyl ions and hydrogen gas to form sodium hydroxide (NaOH, the catholyte, which has detergent properties). This in turn is the alkaline/basic EW that has a pH of 11.5. Newer devices combine the cathode and anode inside one cell to produce a more neutral 8.5 pH solution (16). This neutral solution has low corrosion potential but also a less potent acidic solution due to the lower HOCl concentration in favor of more hypochlorite (ClO^−^). Therefore, some manufacturers recommend adding acid to buffer the solution toward a slightly acidic state, thus increasing the concentration of HOCl (19). In recent years, these EW generator units have become available to individuals and small businesses, and several companies that manufacture electrolytic units have pursued and received EPA registration and US Food and Drug Administration (FDA) approval for use of their products in the food industry and for food decontamination.

As previously noted, acidic EW is the most potent and popular type. Research indicates that HOCl, in addition to its high antimicrobial potency, has maximal effectiveness at low pH because under these conditions the target membrane surfaces are more vulnerable to HOCl attack (27, 28). Therefore, acidic EW is considered a more capable and effective disinfectant than conventional chemical disinfectants such as regular sodium hypochlorite. Studies have shown that acidic EW at an available chlorine concentration of 50 mg/L can reduce bacterial counts by more than 5 logs with a 1-min contact time. By comparison, a solution of sodium hypochlorite required a much higher available chlorine concentration (120 mg/L) to achieve similar inactivation levels (3).

ORP, FAC content, and pH are the major parameters in determining EW potency and efficacy. These parameters can be affected by the build quality of the EW generation unit, the electrode materials and sizes, and the electric current amperage. Additionally, generation conditions such as salt concentration, processing time, and temperature are important to the efficacy of the resulting EW. For example, increasing the initial salt concentration produces higher FAC concentrations. pH, ORP, and FAC concentration are all time- and current-dependent, with higher amperage producing higher ORP and FAC levels, and lower pH (16, 29, 30). Furthermore, the EW unit electrodes have a lifespan, and will deteriorate and corrode over time, especially the anode if it is not made from a noble metal, such as platinum; electrodes in poor condition may not generate the requisite concentration of hypochlorous acid. Thus, the electrolysis process needs to be monitored frequently to ensure the generation of a potent disinfectant. The easily measured parameters FAC concentration and pH can be used for this purpose, as well as being useful indicators of the potency and efficacy of the generated disinfectant (16).

The potential safety, biohazards, and toxic effects of EW have been compared with those of other conventional disinfectants. EW is generally recognized as safe (GRAS) and does not contain substances considered hazardous to health at the concentrations presently used (8). EW is also considered as a natural and green disinfectant because it is environmentally friendly; it contains only water and salt and rapidly decays in the open atmosphere, leading to very low risks to human health and the environment (17). EW is non-irritating to users and not corrosive to the skin, mucous membranes, or other organic materials, unlike other acidic disinfectants, such as hydrochloric acid-based disinfectants. Acidic disinfectants are very irritating to the skin and mucous membranes and can cause eye damage, or even blindness (6, 17). Furthermore, formaldehyde- and glutaraldehyde-based disinfectants are noxious and cytotoxic and may pose a serious health risk in addition to adversely affecting the environment. sodium hypochlorite-based disinfectants have severe adverse effects and cause skin irritation, membrane irritation, and acute toxicity. Quaternary ammonium-based disinfectants irritate the skin and mucous membranes, and are related to the development of airway allergy as well as induce occupational asthma and contact dermatitis (31). For alcohol-based disinfectants and hand sanitizers, frequent use of high-concentration formulations may lead to skin damage or contact dermatitis with skin irritation, dryness, redness, and cracking. They may also cause minimal chronic systemic toxicity via percutaneous absorption and, in cases of accidental ingestion, particularly by children, can cause acute systemic toxicity. This may be life-threatening and cause nausea, vomiting, and varying degrees of respiratory and nervous system depression. Additionally, they may negatively impact aquatic organisms and wildlife (32). EW does not lead to the development of antimicrobial resistance, unlike genotoxic chemicals. However, EW, like other chlorine-based disinfectants, should not be mixed with ammonia-based products as chloramines can be released; in large EW generator units in which a large amount of chlorine gas is emitted, it is recommended to use a standard-type extractor fan to avoid the accumulation of toxic gas (17, 33). EW is not flammable and can be stored without health risks, but it should be kept in closed vessels to preserve its potency by preventing chlorine gas evaporation and contact with the atmosphere (16).

In conclusion, EW satisfies many of the requirements for an ideal disinfectant, including effectiveness against a broad spectrum of pathogens (bactericidal, Fungicidal, and viricidal effects). It reduces cleaning times, is easy to use over large areas, and it has a relatively low operational cost because it can be generated from only salt and electricity by many commercially available units in the clinic. Its disadvantages are few; these include rapid loss of antimicrobial potency due to continual loss of chlorine gas, and significant decreases in potency when in contact with organic matter (either by chemical reactions between chlorine compounds and nitrogen groups in organic matter such as blood and saliva or by blocking the physical access of the disinfectant to the microbial target) (27, 29). EW (particularly the acidic type) is strongly acidic and its free chlorine content is corrosive to some metals during prolonged contact, which can also lead to the degradation of synthetic resin (3, 17). Thus, to overcome these limitations, freshly generated EW should be used or it should be stored in closed vessels to prevent chlorine gas evaporation, maintaining its disinfectant potency. Moreover, EW must be used at higher concentrations when large amounts of blood or body fluids are present, or the area should be pre-cleaned and rinsed with a detergent before applying the EW disinfectant. To prevent metal corrosion and synthetic resin degradation, metal and synthetic resin surfaces should be washed with sterile water after using EW disinfectants (17). This is also supported by previous studies related to the effects of acidic EW on hemodialysis equipment and endoscopes, which showed that the deterioration of metals was within the normal range and no serious corrosive changes occurred after disinfection using acidic EW (34, 35). Therefore, the Japanese Ministry of Health, Labor, and Welfare recommend the use of acidic EW to disinfect endoscopes (6). Other studies reported (17, 36) that EW does not adversely affect stainless steel; therefore, most dental equipment, surfaces, and instruments made from stainless steel can be disinfected using EW.

Despite these few negatives, EW has considerable potential as an alternative surface disinfectant and sanitizer in dental offices, as it meets the many requirements of ideal disinfectants used in these areas. In addition, EW can be produced in the dental office (at low active concentration), which is beneficial in cases of disinfectant shortage, and eliminates the need for and the risks associated with the storage of large amounts of concentrated hazardous cleaning and sanitizing solutions. Disinfectants play a vital role in the continuity of dental provision and safety of dental practices and thus the quality of the services we provide to our patients. Further studies are needed to test the effect of on-site-generated antiseptic mouthwashes based on EW, as previously shown to be effective against different oral bacteria (11), on SARS-CoV-2 inactivation. EW may be useful as a pre-procedural oral rinse, which could have a high impact in breaking the chain of infection in dental office settings by reducing the quantity of virus in spatter and aerosols generated during dental procedures, in conjunction with standard infection control measures such as the use of high volume suction, use of rubber dams, increased Treatment Room and office ventilation, and the strict use of personal protective equipment (37).

## Summary

This report describes the practical steps involved in preparing and quality testing acidic electrolyzed water disinfectant for use in dental offices, notably using affordable and readily available equipment that does not require expert installation. This is clearly of relevance during the current pandemic emergency. This emerging method is of particular importance at present, due to the supply problems experienced by many dentists, and our description is intended to enable dental offices to move quickly and safely toward its use.

## Data Availability Statement

The original contributions presented in the study are included in the article/supplementary material, further inquiries can be directed to the corresponding author/s.

## Author Contributions

All authors listed have made a substantial, direct and intellectual contribution to the work, and approved it for publication.

## Conflict of Interest

The authors declare that the research was conducted in the absence of any commercial or financial relationships that could be construed as a potential conflict of interest.
